# The Therapeutic Role of Ketogenic Diet in Neurological Disorders

**DOI:** 10.3390/nu14091952

**Published:** 2022-05-06

**Authors:** Diana Pietrzak, Kamila Kasperek, Paweł Rękawek, Iwona Piątkowska-Chmiel

**Affiliations:** Chair and Department of Toxicology, Faculty of Pharmacy, Medical University of Lublin, Jaczewskiego 8b Street, 20-090 Lublin, Poland; kamila.kasperek.pharm@gmail.com (K.K.); pabloo0705011@gmail.com (P.R.)

**Keywords:** ketogenic diet, neurological disorders, epilepsy, depression, migraine, Alzheimer’s disease, Parkinson’s disease

## Abstract

The ketogenic diet (KD) is a high-fat, low-carbohydrate and adequate-protein diet that has gained popularity in recent years in the context of neurological diseases (NDs). The complexity of the pathogenesis of these diseases means that effective forms of treatment are still lacking. Conventional therapy is often associated with increasing tolerance and/or drug resistance. Consequently, more effective therapeutic strategies are being sought to increase the effectiveness of available forms of therapy and improve the quality of life of patients. For the moment, it seems that KD can provide therapeutic benefits in patients with neurological problems by effectively controlling the balance between pro- and antioxidant processes and pro-excitatory and inhibitory neurotransmitters, and modulating inflammation or changing the composition of the gut microbiome. In this review we evaluated the potential therapeutic efficacy of KD in epilepsy, depression, migraine, Alzheimer’s disease and Parkinson’s disease. In our opinion, KD should be considered as an adjuvant therapeutic option for some neurological diseases.

## 1. Ketogenic Diet

The ketogenic diet (KD) is a high-fat, adequate-protein, low-carbohydrate diet [1]. Such changes in the proportion of macronutrients lead to glucose sparing and enhanced ketogenesis [2]. This metabolic state is known as “nutritional ketosis”. More and more research shows that the ketogenic diet can have a positive effect on brain functions and peripheral organs, and thus provide therapeutic benefits to a wide range of neurological conditions [3,4]. Although the molecular mechanisms of action of the ketogenic diet are unclear, growing research suggests that KD can be an important element in adjunctive therapy in the treatment of central nervous system (CNS) diseases. More recently, it has been shown that KD can affect the course of diseases by modulating inflammation [5,6,7,8,9,10], controlling the balance between pro- and antioxidant processes [11,12,13,14] and/or altering the composition of the gut microbiome [15].

### 1.1. History of the Ketogenic Diet

The ketogenic diet has its origins in fasting, which has been used since ancient times to treat epilepsy. In 1921, Woodyatt discovered that both starvation and a high-fat diet lead to a state of ketosis. In the same year, KD was implemented at the Mayo Clinic as a treatment for epilepsy by Russell Wilder [16]. At the time, it was considered that more than half of all children suffering from epilepsy improved their condition. This diet was widely used in this group of patients, until the discovery of the first epilepsy drug, diphenylhydantoin (1938) [16].

At the end of the last century, scientists renewed their interest in KD for its potential role in neurological disorders. Vining et al. [17] studied the efficacy of KD in reducing seizures in children (aged 1–8 years) so far unresponsive for treatment with two anticonvulsant drugs. After one year of following the diet, 40% of patients noted a reduction in seizures by more than 50% and 10% of patients showed a complete absence of seizures. A study conducted by Freeman et al. [18] on 150 patients aged 1–16 years with drug-resistant epilepsy confirmed neuroprotective effects of KD. The researchers observed that after one year following the diet, 27% of patients experienced a reduction in seizures of more than 90%. After 10 years, Neal et al. [19] conducted a randomized controlled trial that confirmed the important role of KD in the control of epileptic seizures. After 3 months of dieting, a decrease in seizures of over 50% in 38% of patients aged 2–16 years was observed.

Furthermore, recent research suggests that KD may have a favourable effect on the course of other neurological diseases, including Alzheimer’s disease (AD) and Parkinson’s disease (PD) [20,21,22].

### 1.2. Types and Characteristics of Ketogenic Diets

**Figure 1:**
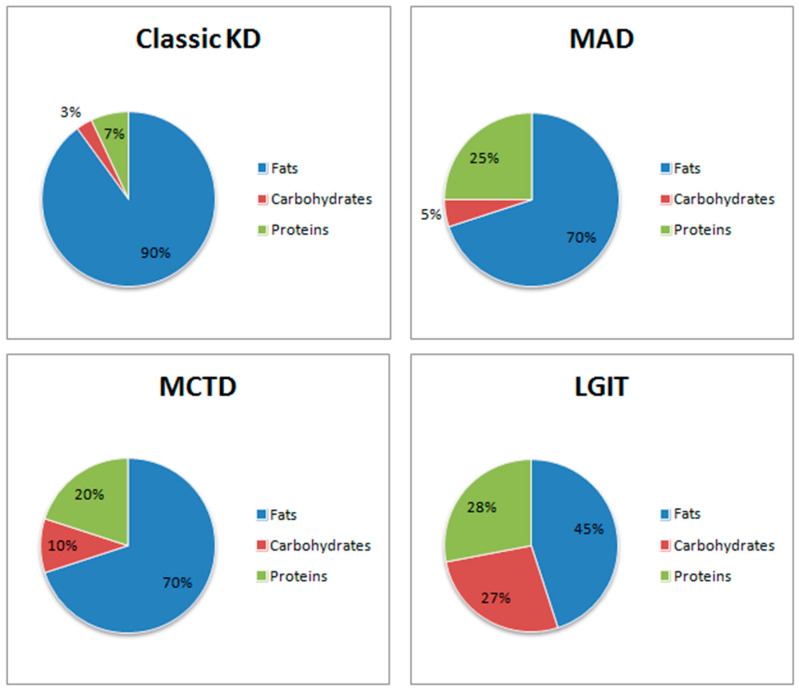
Comparison of selected modifications of the ketogenic diet and their macronutrient ratios. Classic KD- Classic Ketogenic Diet; MAD- High-Protein Ketogenic Diet; MCTD- Medium-Chain Triglycerides Diet; LGIT- Low Glycaemic Index Treatment.

Nowadays, there are many types of ketogenic diet, varying in the proportions of macronutrients, which allows the diet to be tailored to the specific needs of the patient. In Figure 1 was showed the comparison of selected modifications of the ketogenic diet and their macronutrient ratios.

#### 1.2.1. Classic Ketogenic Diet (CKD)

The classic ketogenic diet is characterized by a high dietary fat content, moderate protein intake and low carbohydrate intake, with a macronutrient ratio of 4:1 [23]. The ketogenic diet limits carbohydrate intake to 10% of total daily caloric intake. It follows that a person with a daily energy requirement of 2000 kcal can consume up to 50 g of carbohydrates. However, in the initial phase of diet, carbohydrates should be limited to about 20 g per day. Such a low carbohydrate supply ensures that the body adapts and redirects the metabolism to use fatty acids as the main source of energy.

#### 1.2.2. Modifications of Classic Ketogenic Diet

##### High-Protein Ketogenic Diet (MAD)

The high-protein ketogenic diet is also known as the Modified Atkins Diet (MAD) [24]. The induction phase lasts indefinitely and during this phase the carbohydrate intake is no more than 20 g per day. MAD assumes that the ratio of fats to carbohydrates and protein together is 1–2:1 [24,25]. This diet does not involve limiting the amount of protein or calories consumed, making it easier to maintain and also easier to manage [25].

##### Medium-Chain Triglycerides Diet (MCTD)

MTCD is a type of KD where medium-chain triglycerides (MTC) are predominant [26]. MTCD provides faster absorption of the triglycerides into the bloodstream. The substitution of long-chain fatty acids for short-chain fatty acids, which are metabolised faster, results in obtaining more ketone bodies per kilocalorie. Higher efficiency of this process results in a lower requirement for fats, hence making it possible to consume larger amounts of carbohydrates and proteins [27]. This is a fundamental difference which determines the long-term maintenance of the diet, as it is less strict than classic KD [28,29]. In addition, this type of diet improves mitochondrial function [30].

##### Very Low Calorie Ketogenic Diet (VLCKD)

Carbohydrate intake on this diet varies between 20–50 g per day or may be less than 10% on a 2000 kcal per day [31]. Due to individual differences, not every patient can achieve ketosis with these macronutrient ratios. This modification can be used as an induction to ketogenic diets with higher protein content.

##### Low Glycaemic Index Treatment (LGIT)

Low Glycaemic Index Treatment (LGIT) is an alternative to the ketogenic diet. It is a high-fat diet in which replacement of high glycaemic index (GI) foods with low-GI foods is fundamental. The GI indicates how much food raises blood glucose levels compared to the same amount of reference carbohydrates [32]. Although this diet does not lead to continuous ketosis, it has a positive effect on carbohydrate metabolism. It is easier for patients to maintain and is therefore popular with younger patients during hospitalization [1].

##### Cyclical Ketogenic Diet (CKD)

This consists of cyclical periods of a classic ketogenic diet and a high carbohydrate diet (with 45–65% carbohydrates). The latter is aimed at replenishing glycogen stores in the muscles [33].

##### Targeted Ketogenic Diet (TKD)

This type of ketogenic diet allows a person to consume more carbohydrates around intense physical activity to maintain performance while not affecting the state of ketosis [33].

## 2. Metabolic Alterations in the Brain Associated with the Ketogenic Diet

Although the brain makes up only about 2% of body weight, it is the most energy-intensive organ in the body. It is known that not only glucose can be a source of energy for the brain, but also ketones which can meet up to 60% of the brain’s total energy needs [34]. An increasing amount of research shows that the ketogenic diet by biochemical pathway changes can have a positive effect on brain functions and may deliver therapeutic benefits to a wide range of neurological conditions.

**Figure 2:**
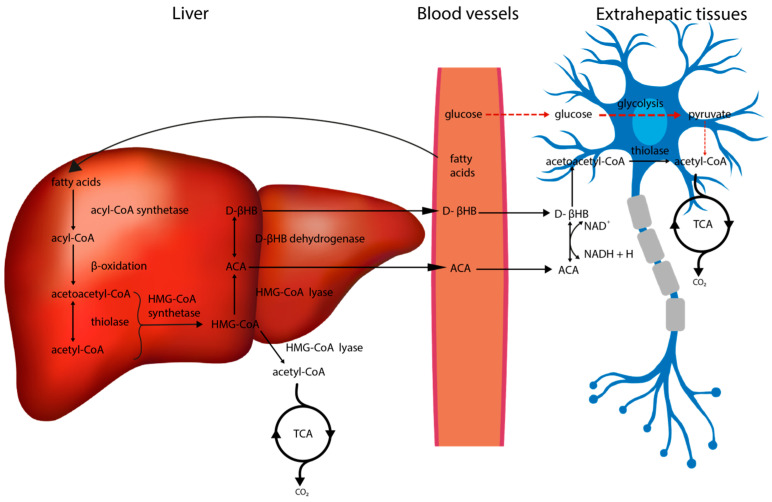
Ketogenesis. Fatty acids undergo the process of β-oxidation in the liver, resulting in the formation of ketone bodies. These are then transported to the blood vessels and then to the neurons where, after conversion to acetyl-CoA, they enter the TCA cycle. The alternative energy source compensates for the smaller contribution of glucose to the ATP yield. (HMG-CoA: 3-hydroxy-3-methyl glutaryl-CoA; HMG-CoA synthetase: 3-hydroxy-3-methyl glutaryl-CoA synthetase; HMG-CoA lyase: 3-hydroxy-3-methyl glutaryl-CoA lyase; ACA: acetoacetate; D-βHB: D-β-hydroxybutyrate; TCA: tricarboxylic acid cycle).

The aim of a fat-rich diet is to induce a state of ketosis in the body, characterised by increased lipolysis as well as ketogenesis [35]. As shown in Figure 2 the fatty acids are intensively oxidized in the liver, resulting in the formation of significant amounts of ketone bodies (KBs), such as acetoacetate (ACA), D(-)3-hydroxybutyrate (D-βHB, β-HB) and acetone. The first two can enter the citric acid cycle (tricarboxylic acid cycle, TCA cycle) and be used to obtain ATP by neurons.

Leino et al. [36] demonstrated that the level of monocarboxyl transporter (MCT), which is responsible for KBs transport across the blood–brain barrier (BBB), is increased in animals fed KD diet compared to controls fed a predominantly carbohydrate diet. Increased transport of ketone bodies was also confirmed by Bentourkia et al. [37] using positron emission tomography (PET) and ^11^C-labeled ACA. This study demonstrated about a seven- to eightfold increase in ^11^C-ACA brain uptake in a state of ketosis induced by KD or starvation, compared to controls fed a carbohydrate-rich diet. Subsequently, the produced KBs are converted to acetyl-CoA in extrahepatic tissues and participate in the citric acid cycle as an energy source [38]. As shown in animal experiments, the obtaining of energy from ketone bodies has many benefits that relate to the nervous system functions. Among others, their transformation increases the total energy pool, which is used by neurons to produce neurotransmitters [39].

Interestingly, β-HB inhibits lipolysis, thus controlling haemostasis and ketogenesis [5], through activation of hydroxycarboxylic acid receptor 2 (HCA2, PUMA-G, GPR109A). Mice lacking this receptor show a higher intensity of lipolysis and ketogenesis, indicating that this receptor is essential for the inhibition of lipolysis by β-HB [40].

The latest research shows that KD can change the ratio of NAD^+^/NADH, increasing the availability of NAD^+^ in the brain, which has a significant impact on cellular pathways involved in inflammatory response, DNA damage repair, and circadian rhythm regulation [14,41]. This makes the ketogenic diet potentially capable of alleviating the symptoms of diseases of which pathogenesis is related to the previously mentioned processes.

### 2.1. The Impact of the Ketogenic Diet on Glucose Metabolism

When the supply of carbohydrates from the diet is insufficient, the brain acquires energy through ketogenesis. Multiple studies indicate that the shift of brain metabolism from glucose oxidation to ketone bodies utilization requires adaptation [42,43,44]. Once the organism has adapted to using KBs as the main source of energy, they can cover up to 60–70% of the energy required for proper function of the brain [34,45]. A study and meta-analysis conducted by Zhang et al. [46] proved that the rate of brain adaptation depends on the duration as well as the severity of ketosis.

The Zilberter et al. [2] analysis concluded that KBs act by sparing glucose rather than inhibiting glycolysis. Due to this mechanism, glucose performs other functions in which it cannot be replaced, i.e., biosynthesis of other compounds, such as alanine [47] and glutamate [48], glycogenesis, and antioxidant protection [2].

Glucose is transported in the brain by three isoforms of glucose transporters (GLUT): (1) 55 kDa GLUT 1, expressed by endothelial cells, (2) 45 kDa GLUT 1, expressed by astrocytes, (3) GLUT 3, produced by neurons [49]. Leino et al. [36] revealed that GLUT1 levels are elevated in endothelial cells and neuropil of rats put into ketosis, compared to a group fed a high carbohydrate diet. Enhanced glucose transport to the brain may also be associated with the action of insulin-like growth factor (IGF1). Rats fed a ketogenic diet with energy restriction showed increased expression of (1) insulin-like growth factor IGF1 receptors in every part of the brain, (2) IGF1 binding protein, which inhibits IGF1 proteolysis, in Purkinje cells, and (3) GLUT 1, GLUT 3 mRNA [50]. Experimental research on the effects of IGF1 on the mice brain has shown that it acts synergistically with insulin on the translocation of the glucose transporter GLUT1 to the astrocyte cell membrane, contributing to enhanced transport of glucose to the brain [51].

At the same time, astrocyte metabolism has been shown to be augmented by KD [52]. Astrocytes activate glycolysis and glycogenolysis, which provide energy to maintain essential functions of these cells, such as the removal of excess glutamate and K^+^ ions from the synaptic cleft [53].

### 2.2. The Impact of the Ketogenic Diet on Amino Acid Metabolism and Neurotransmitter Synthesis; Glutamate-Glutamine Cycle

Glutamate is an excitatory amino acid and therefore its concentration in the synaptic gap must be kept at a low level. It is transported via vesicular glutamate transporters (VGLUTs). Their action is dependent on Cl^−^ ions, which act as allosteric modulators. Absence of these ions inhibits glutamatergic transport [54]. Juge et al. [55] demonstrated that ketone bodies cause reversible inhibition of glutamate transport in hippocampal neurons by binding to the Cl^−^ ion site. ACA exhibited a stronger effect than other intermediates of cell metabolism, such as β-HB and pyruvate.

**Figure 3:**
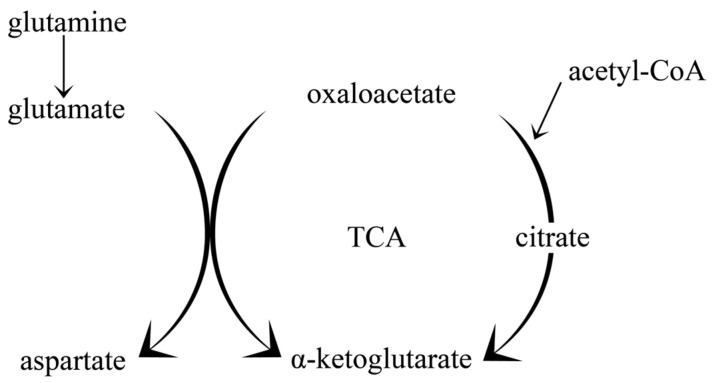
Amino acid metabolism in the brain. During ketosis, the amount of glutamate is increased. This may contribute to the increased flow through the TCA cycle.

Long-term consumption of a fat-based diet with a concomitant reduction in carbohydrates increases the flow through the TCA cycle via amplified production of acetyl-CoA, and enhanced activity of the acetyl-CoA reaction with oxaloacetate [52]. The pathway for obtaining glutamate is also intensified due to the reduced availability of oxaloacetate compared to the physiological state, as a result of the increased utilisation of this compound in the reaction with acetyl-CoA in the citric acid cycle [52]. This causes reduced conversion of glutamate to aspartate in the reaction glutamate + oxaloacetate = aspartate + α-ketoglutarate [56] (see Figure 3).

Studies on ketonic mice have shown that leucine concentrations were higher in both blood and forebrain of these animals, while glutamate and glutamine concentrations were not different compared to controls fed a predominantly carbohydrate diet [56]. This may result in the increased transport of glutamate by astrocytes to neurons, which requires the delivery of an ammonia molecule [57], mostly generated from leucine [58]. These studies indicate an enlargement of the available glutamate pool that can favour synthesis of the inhibitory neurotransmitter gamma-aminobutyric acid (GABA), as confirmed by studies on synaptosomes using high concentrations of ACA [59]. Moreover, studies using ^13^C-labeled glucose and acetate revealed that the carbon found in GABA in the ketosis state was derived from acetate [52]. An increased GABA/glutamine ratio was also observed.

### 2.3. The Impact of the Ketogenic Diet on Insulin Signalling

Insulin is a hormone produced by pancreatic β cells that increases glucose uptake by the cells, thereby reducing blood glucose levels [60]. The lack of tissue sensitivity to insulin, that is, insulin resistance, as well as defective secretion of this hormone is associated with type 2 diabetes mellitus (T2DM), defined now as a pandemic of the 21st century [60,61,62,63]. Nevertheless, its action is not limited to peripheral tissues. Insulin crosses the blood–brain barrier and binds to insulin receptors (IRs) in the brain, resulting in the activation of signalling pathways [64]. However, certain structures in the brain, like the hypothalamus, are more susceptible to its action due to the absence of the BBB, which allows insulin to pass more freely [65]. The PI3K/Akt cascade is one of the main signalling pathways activated by insulin [64]. It may subsequently activate other pathways such as mTORC1, GSK3β and FoxO transcription factors, which are involved in many neuronal functions [66]. These pathways also have the potential to lead to the death of neurons, via the removal of damaged proteins or increased phosphorylation of tau proteins, being one of the pathologies observed in Alzheimer’s disease [67].

There is limited information on the impact of KD on insulin signalling in the brain. Some research shows that insulin can regulate the secretion of neurotrophic factors and neurotransmitters and also interact with the gastrointestinal microbiome [15]. Gupta et al. [68] focused on the possible antidepressant effect of insulin on the disrupted neurotransmitter system in diabetes. Insulin administration to mice with streptozocin-induced diabetes elicited higher mouse scores in forced swim test, tail suspension test and spontaneous locomotor activity compared to healthy mice. In addition, the diabetic mice showed higher serotonin levels and reduced monoamine oxidase (MAO) A and B activity in the brain.

Taking into consideration that impaired insulin signalling in the brain is heavily associated with Alzheimer’s disease [69,70,71,72,73,74], affecting levels of this hormone may improve a patient’s condition. Studies in the last few years confirm that KD enhances insulin responsiveness and reduces fluctuations in glucose levels [75,76,77]. This effect is manifested by higher scores on cognitive tests, namely the Montreal Cognitive Assessment, suggesting that KD may have a significant influence on alleviating insulin resistance in the brain [75,76]. Case studies of subjects suffering from Alzheimer’s disease (heterozygous ApoEɛ4 carriers) reported by Stoykovich et al. [75] and Morrill et al. [76] demonstrated that treatment with KD for 10 months reduced (1) fasting glucose levels by 24–25%, (2) fasting insulin by 67–85.3%, (3) homeostatic model assessment for insulin resistance (HOMA-IR) by 75–88.8%, respectively. Furthermore, a randomised controlled trial conducted by Fortier et al. [77] showed that administration of ketogenic drinks to patients with mild cognitive deficits for 6 months resulted in improved episode memory, language skills and executive function.

### 2.4. The Impact of the Ketogenic Diet on Oxidative Stress

Products of cellular respiration, reactive oxygen species (ROS) and reactive nitrogen species (RNS), are highly reactive and when their detoxification is decreased, they may cause lipid peroxidation, cell membrane, DNA and protein damage [78]. Imbalance between the production of ROS and RNS and their insufficient neutralization is defined as oxidative stress. It appears to play a pivotal role in the pathogenesis of neurodegenerative diseases such as Alzheimer’s disease and Parkinson’s disease [79,80]. Many in vitro and animal studies confirm the beneficial effects of a ketogenic diet and ketone bodies, by enhancing free radical scavenging and improving activity of antioxidant systems [11,12,13,14].

In vitro administration of ACA and β-HB to HT22 cell lines and hippocampal neurons with glutamate-induced oxidative stress increased their viability [11]. In the study of Maalouf et al. [12], administration of ACA and β-HB to neocortical neurons and isolated mitochondria derived from these cells decreased ROS production and the associated increased NADH oxidation. Reduced cell death was also observed. Sullivan et al. [13] observed that oligomycin (ATP-synthase inhibitor) induced ROS production was lower in KD-fed mice compared to mice fed a standard diet. At the same time, uncoupling protein 2, 4 and 5 (UCP 2, UCP4 and UCP5) levels were higher in KD-fed mice, resulting in increased maximum mitochondrial respiration rates. Hasan-Olive et al. [14] also found that mice with uracil-DNA-glycolase 1 enzyme mutation, which caused mitochondrial toxicity, exhibited higher UCP2 levels in hippocampal CA1 neurons when fed KD, probably due to upregulation of PGC1α-SIRT3-UCP2 axis, caused by β-HB. This study also showed increased oxygen consumption and amplified NAD^+^/NADH ratio in rats’ hippocampal neurons and human fibroblasts cell lines, with H_2_O_2_-induced oxidative stress.

### 2.5. The Impact of the Ketogenic Diet on Neuroinflammation

Recent studies imply that neuroinflammation can be not only a concomitant symptom of nervous system diseases such as epilepsy, multiple sclerosis, migraine, Alzheimer’s disease (AD) or Parkinson’s disease (PD), but also an important factor of their development [69,81]. Neuroinflammation is associated with microglia activation and increased release of inflammatory factors such as tumour necrosis factor (TNF), interleukins (IL-1β, IL-6) and free radicals, which can result in progressive dysfunction or cell death in the brain [39,82]. Studies on animal models of Parkinson’s disease demonstrated that KD can reduce inflammation in CNS by decreasing the microglial activation and reducing expression of pro-inflammatory cytokines [6,83].

Besides, it was noticed that KD supports anti-inflammatory and antioxidant factors production which in addition favours limitation of inflammation in CNS [84]. Previous studies showed that β-HB (produced in increased amounts in KD) inhibited the inflammatory response by up-regulation of anti-inflammatory genes such as NF-κBIA, MAP3K8 and TLR5 and down-regulation of pro-inflammatory genes such as TNFSF6, TNF-α, and nuclear factor-ĸB (NF-κB) [6,7,8,9,10].

Yang et al. [83] showed that KD significantly reduces levels of inflammatory factors such as IL-1β, IL-6 and TNF-α in substantia nigra and reduces microglia activation in an animal model of Parkinson’s disease induced by the administration of 1-methyl-4-phenyl-1,2,3,6-tetrahydropyridine (MPTP). Rodents exhibited reduced inflammation, enhanced dopaminergic transmission in the substantia nigra and improved motor function. After MPTP injections, KD-fed mice scored twice as high on the rota-rod motor coordination test as mice fed the standard diet.

As previously mentioned, Taggart et al. [40] showed that β-HB is an endogenous ligand of HCA2 receptor and its action is similar to nicotinic acid. A study by Zandi-Nejad et al. [85] on lipopolysaccharide-induced inflammation (LPS) in murine bone marrow-derived macrophages showed that stimulation of the HCA2 receptor by nicotinic acid inhibits the production of pro-inflammatory cytokines through NF-ĸB signalling pathways. In turn, inhibition of NF-ĸB down-regulates two genes key to the inflammatory response, COX2 and enzymes involved in nitric oxide synthesis [86]. A study by Fu et al. [6] in rats with model Parkinson’s disease induced by LPS administration to the substantia nigra also showed a neuroprotective effect of β-HB on dopaminergic neurons, as well as a reduction in microglia activity. In vitro studies confirmed that these actions were due to HCA2 receptor activation.

Shimazu et al. [7] noted that β-HB inhibits histone deacetylases 1, 3, 4 (HDAC 1, HDAC 3, HDAC 4) in vitro. ACA also shows inhibitory activity against HDAC class I and IIa, but at concentrations that are not attainable by nutritional ketosis. Increased histone acetylation results in up-regulation of antioxidant systems, including the FOXO3A network and metallothionein 2. Increased FOXO3 expression causes increase in Mn-SOD and catalase levels [87]. Pump administration of ketone bodies to the kidney of mice resulted in reduced lipid peroxidation and protein carbonylation in the kidney, compared to a control group fed a standard diet. Inhibition of HDACs also increases the activity of antioxidant systems by increasing PPAR-α activity [7,88]. A study by Huang et al. [9] showed that β-HB induces macrophage adaptation to anti-inflammatory morphology through promotion of ramification and pro-phagocytic effects. This is due to the enhancement of the protein kinase B (Akt)-small RhoGTPase axis, which can occur through the inhibition of HDACs.

Studies in mouse models of inflammatory diseases by Youm et al. [8] showed that β-HB inhibits the decrease in cytoplasm potassium ions and thus pyrin domain-containing 3 inflammasome (NLPR3) activity. The mechanism of this action is not fully elucidated, but it is suggested that it may be related to calcium signalling. This hypothesis is supported by the study of Lee et al. [89], which showed that the Ca^2+^-sensing receptor is involved in NLPR3 activation in mice. NLPR3 inflammasome is considered to be one of the units linking the immune system and inflammatory responses. It is a multiprotein complex, secreted mainly by immune cells, in response to a decrease in potassium ion levels in the cytoplasm. This results in activation of caspase-1 (which converts IL-1β to its active form) and production of the pro-inflammatory cytokines IL-1β and IL-18 in macrophages [90]. Shao et al. [91] proposed inflammasome NLPR3 inhibition as a potential therapy in AD, PD, multiple sclerosis and depression.

### 2.6. The Impact of the Ketogenic Diet on Brain-Derived Neurotrophic Factor (BDNF)

As is known, brain-derived neurotrophic factor (BDNF) has beneficial effects on neuroprotection and neuroregeneration of cells. BDNF belongs to a group of proteins that support CNS function, namely neurotrophins (NTs). NTs are synthesized mainly in CNS, but also in T and B lymphocytes, monocytes, smooth muscle cells and skeletal muscle cells, as well as in endothelium of blood vessels [92]. BDNF influences the development of the nervous system. It enables processes of cell differentiation, neuronal development, improves growth and survival of neurons, favourably influences the efficiency of neurogenesis, synaptogenesis and synaptic plasticity [93]. Histone deacetylase inhibition contributes to the stimulation of BDNF secretory processes in cortical neurons.

KBs formed with KD inhibit histone deacetylase and thus increase BDNF secretion. β-HB stimulates BNDF gene expression, which increases BDNF protein levels in cortical neurons. This is done through activation of the BDNF gene promoter IV and a mechanism involving the transcription factor NF-κB and the histone acetyltransferase p300. This is an extremely important property to limit the progression of neurodegenerative changes [94,95].

### 2.7. The Impact of the Ketogenic Diet on Activity of ATP-sensitive Potassium Channels

Bearing in mind the fact that the largest amount of potassium channels is located in the brain, it makes it the most susceptible to changes of their activity and the associated disturbances of central nervous system functions [96]. ATP-sensitive potassium channels (K_ATP_) are a subtype closely related to cellular metabolism [96] and linked to electrical activity [97]. A decreasing ATP/ADP ratio induces the opening of these channels, while increasing ATP levels result in their closing. Their activation is known to exert protective effects against oxidative stress by reducing ROS production and improving mitochondrial metabolism [98].

The substantia nigra pars reticulata (SNr) and the subthalamic nucleus (SN) are both abundant in K_ATP_ channels [99]. They belong to the basal ganglia, involved in the control of movement [100] and have been considered as a seizure gate [101]. Impairment of these structures is associated with epilepsy and Parkinson’s disease [102]. It has been noted that frequent spontaneous firing of GABA-ergic neurons in the SNr can induce seizures [103]. Inhibition of these neurons, on the other hand, hinders the onset of convulsions. Both β-HB and ACA have been shown to reduce the frequency of neuronal firing in SNr in brains of mice [104,105]. This may be related to a reduction in the importance of glycolysis in metabolism under ketosis and consequently a decrease in glycolisis ATP production, which in turn results in K_ATP_ activation and reduced excitability. Furthermore, β-HB increases the probability of K_ATP_ channels opening in the hippocampus, which may help granule cells to maintain seizure-gate activity and prevent convulsions [106].

Juge et al. [55] demonstrated that administration of ACA reduced seizure intensity and decreased glutamate secretion, while having no effect on dopamine levels in rat with seizures induced by 4-aminopyridine (potassium channel blocker). However, this effect was reversible: after ACA removal, the effect of 4-aminopyridine intensified.

Kim et al. [107] attempted to determine the type of channels involved in reducing metabolic stress. For this purpose, the researchers investigated the effects of β-HB and ACA on K_ATP_ channels located in rat and mouse hippocampi. The results confirmed the protective effect of KBs, induced by activation of ATP-dependent potassium channels against oxidative stress. The researchers noted that blocking mitochondrial K_ATP_ channels with 5-hydroxydecanoate and absence of plasmalemmal K_ATP_ channels abolished the neuroprotective effect of KBs, thereby indicating that the neuroprotective effect is obtained by affecting both types of channels.

### 2.8. The Impact of the Ketogenic Diet on Beta Amyloid and Tau Protein Synthesis

The processes leading to the development of AD are inextricably linked to abnormal transformations of beta amyloid (Aβ) and tau protein as a result of which pathological conglomerates of these structures are formed. At the root of this process is the dysfunctional activity of mitochondria. This entails a decrease in the level of energy from glucose metabolism and an increase in the accumulation of tau protein and Aβ [26]. Given the complex nature of AD aetiology and the positive effects of KD in older patients diagnosed with AD, there is a justification for the wide use of KD in neurodegenerative diseases [108].

According to studies, KD may contribute to reducing the level of accumulation of beta amyloid, and reversing its toxicity, by affecting the neuropathological and biochemical processes that are found in AD [20,26]. It has been proven that KD can reduce the volume of pathological beta aggregates of amyloid and tau protein in brain homogenates of laboratory animals [78]. In mice treated with a ketogenic diet for 40 days, there was a 25% reduction in amyloid beta deposits with no effect on the ability to recognize simple objects [20,26]. Resembling experiment conducted over 43 days showed similar effects, but scientists did not verify an effect of diet on the cognitive abilities of animals [109]. The reason for this, most likely, was too short duration of this treatment. Diet-derived ketone bodies might help to improve memory and cognitive function. The participation of KD in reducing the level of risk of developing the disease by improving the function of cerebral circulation and improving the metabolic actions (including lowering glucose levels and augmenting the intestinal microflora) was found [78].

To sum up, the role of the ketogenic diet in the treatment and prevention of Alzheimer’s disease seems to be growing recently. The diet itself has an impact on many metabolic processes important in AD and other neurodegenerative diseases as well. Currently, it is believed that the positive effect on cognitive, metabolic and biochemical functions depends on the length of maintaining high levels of ketone bodies in the blood [26]. It is difficult to determine the importance of KD in the treatment of neurodegenerative diseases in the future, if only because of the limitations in the use of this diet in older patients with particular comorbidities [78]. Despite the lack of definition of precise mechanisms determining the action of KD in neurodegenerative diseases and the lack of consistency in the results of independent laboratory tests, it seems worthwhile to continue research determining the exact mechanisms behind the improvement of patients’ condition [26]. It is equally important to determine the long-term impact of KD on the overall well-being of patients using it [78].

## 3. The Impact of the Ketogenic Diet on Gut Microbiota

Recently, the influence of the gut microbiota has been increasingly studied in the context of neurological diseases [110,111,112,113]. However, it is not known whether changes in the composition of the microbiota are a cause or an effect of neurological disorders. The gut microbiota–brain axis involves a bidirectional flow of information between the two organs. The brain affects the gut through norepinephrine release, which modulates conditions in the intestine [114]. The gut microbiota, on the other hand, influence central nervous system through the vagus nerve and via bioactive substances, such as short chain fatty acids (SCFAs), tryptophan derivatives and secondary bile acids. The composition of the microbiota is constant throughout most of an adult’s life and it is generated by, among other things, individual lifestyle, eating habits or health conditions [115]. Certain types of bacteria that inhabit the large intestine are crucial for the proper functioning of the human body, due to the synthesis of K- and B-group vitamins, as well as the synthesis of neurotransmitters [116,117]. SCFAs (acetate, propionate and butyrate) produced from indigestible carbohydrates, by the strains of *Firmicutes* and *Bacteroidetes* along with *Bifidobacteria*, are not only a source of energy for colonocytes, but also exhibit a variety of beneficial actions for the human organism [118,119]. High concentrations of SCFAs in the intestinal lumen inhibit the growth of Gram-negative bacteria of the *Enterobacteriaceae* family [120]. These bacteria, through the production of LPS, can lead to inflammation [113,121]. Hence, inhibiting their growth may indirectly supress the inflammatory process. In addition, butyrate exhibits anti-inflammatory and oxidative stress modulating effects via inhibition of NF-κB activation, histone deacetylases and up-regulation of peroxisome proliferator activated receptor γ (PPAR- γ) [122].

Many studies show that the effect of probiotics, prebiotics, synbiotics and antibiotics may have an effect on the course of diseases such as Parkinson’s disease, depression, and Alzheimer’s disease, confirming the vital impact of the gut microbiota composition on the proper nervous system functioning [113,123,124,125]. Studies also suggest that a favourable microbiota profile may be possible during KD, which results in an alleviation of epilepsy symptoms [126,127,128,129,130]. This may be related to the beneficial effects of certain bacteria inhabiting the large intestine on inflammation, or rebalancing of neurotransmitter systems.

Although the hypothesis of the involvement of the microbiota in the pathogenesis of many diseases has been established for some time, studies on the effects of the ketogenic diet on the composition of the gut microbiota have appeared only relatively recently. The available research shows that the ketogenic diet affects the microbiota in a specific manner regardless of disease [131]. A systematic review conducted by Paoli et al. [131] demonstrates that a ketogenic diet increases *Bacteroides*, *Prevotella* and decreases *Firmicutes* and *Actinobacteria* strains in patients suffering from epilepsy. These adjustments in the composition of the gut microbiota resulted in a reduction in seizure frequency by over 50% and severity in more than 50% patients. Clinical trials showed that a very low-calorie ketogenic diet in obese patients with insulin resistance resulted in increased *Bacteroides* and decreased *Firmicutes* [132,133]. Both studies reported significant weight loss among patients and improvements in the tested parameters, i.e., reductions in fasting glucose, insulin, HOMA-IR, blood pressure and low-density lipoproteins. Additionally, Basciani et al. [132] showed that changes in microbiota composition were dependent on protein source; whey protein exhibited the strongest increase in *Bacteroides* and decrease in *Firmicutes*, compared to plant- and animal-derived protein. However, the effect of KD on *Bifidobacterium*, which belongs to the *Actinobacteria* phylum, remain inconclusive. After 1 week of implementation of KD in infants, Xie et al. [126] reported increase in *Bifidobacterium*, while the study by Ang et al. [133] on adult overweight men fed KD for 4 weeks showed a reduction in *Bifidobacterium*, which resulted in a decrease in pro-inflammatory Th17 cells. This may be a result of many variables, including the age of the studied patients, as well as the products they consumed during the diet.

Probiotics are defined as beneficial bacterial strains, while prebiotics refer to non-digestible substances that stimulate the growth of these bacteria [134]. A synbiotic combines the two previously mentioned terms. Probiotic bacteria include bacteria such as *Lactobacillus*, *Bifidobacterium* or *Akkermansia muciniphila*. As mentioned above, the effect of KD on the number of *Bifidobacterium* is not fully determined, whereas an increase in both *Lactobacillus* and *Akkermansia muciniphila* was reported in KD-fed mice [135,136]. These observations resulted in a reduction in seizure frequency [136], as well as a reduction in AD risk by improving blood vessel function in the brain [135]. The neuroprotective effects of bacterial strains are assumed to be related to the anti-inflammatory effects of probiotic bacteria, as well as the reduction of intestinal permeability and the associated impeded translocation of bacteria [135,136,137].

Supplementation with prebiotics, probiotics or synbiotics during KD has not been explored thoroughly. Eor et al. [138] investigated the effects of KD, probiotics and synbiotics in mouse models of epilepsy. Administration of a probiotic (*Lactobacillus fermentum*) as well as a synbiotic (*L. fermentum* with galactooligosaccharide) to mice consuming KD significantly reduced the number of seizures. The ketogenic diet itself delayed seizure onset considerably longer compared to the other KD-fed groups, as well as mice fed a normal diet. Furthermore, the results of the experiment indicate a beneficial effect of synbiotic supplementation on lipid profile over the course of KD. Mice receiving the synbiotic and fed a KD exhibited reduced levels of triglycerides, as well as cholesterol. In addition, they showed the highest levels of GABA. In contrast, Mu et al. [139] observed no effect of probiotics (*Streptococcus thermophilus*, *Lactococcus lactis* subsp. *lactis*) on the anticonvulsant effect of the diet. The administration of probiotics to mice reduced the lipid disturbance caused by the high-fat diet through effects on AMPK signalling and stimulation of lipid oxidation.

Although the number of conducted studies is still limited, their results are promising. More research is needed to determine the validity of using pre-, pro- and synbiotics in combination with KD. Current knowledge suggests, however, that additional enrichment of the microbiota may not affect the course of neurological disease itself, but may have a beneficial effect on the side effects of the ketogenic diet.

## 4. Etiopathogenesis of Neurological Diseases and Therapeutic Role of Ketogenic Diet

**Figure 4:**
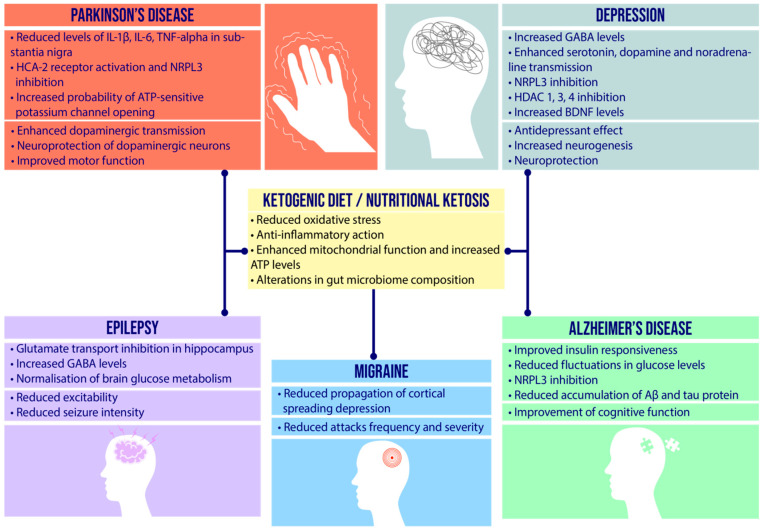
The potential role of the ketogenic diet/nutritional ketosis in neurological disorders. The large amount of ketone bodies formed during a low-carbohydrate, high-fat diet may have a beneficial effect on many of the pathological processes found in neurological diseases, thus potentially offering beneficial adjuvant therapy. (IL-1β: interleukin 1β; IL-6: interleukin 6; TNF-alpha: tumour necrosis factor alpha; HCA-2: hydroxycarboxylic acid 2; NRPL: NOD-like receptor family pyrin domain containing 3 inflammasome; HDAC: histone deacetylases; BDNF: brain-derived neurotrophic factor; GABA: gamma-aminobutyric acid; Aβ: amyloid β).

Currently, there is a consensus among researchers to determine the leading causes of neurological diseases. It is believed that serious disease states as well as transient disease symptoms are results of excessive expression of reactive oxygen forms and pro-inflammatory factors. Long-term and progressive oxidative stress contributes to the destruction of hippocampal cells and a decrease in BDNF production, which contributes to the weakening of nerve cells and the formation of brain lesions. In recent years, more and more evidence has been discovered in favour of the fundamental role of oxidative stress in the disruption of metabolic, ischemic and inflammatory processes in nervous tissue [140]. These phenomena can significantly affect the structure and functions of nervous tissue, which is extremely susceptible to this type of process [141]. In recent years, the microbiome–gut–brain axis has become increasingly important in the pathogenesis of neurological diseases [113,142,143]. By affecting inflammatory pathways, as well as synthesizing specific compounds, the composition of the microbiota may influence the development and also the inhibition of disease progression, and may represent another potential therapeutic strategy for neurological disorders. The complexity of the pathogenesis of CNS diseases means available therapies have little effect; therefore, the multi-target nature of the ketogenic diet makes it an attractive complementary therapy that may enhance the efficacy of administered pharmacotherapy or alleviate symptoms in drug-resistant disease entities. In Figure 4 was showed the potential role of the ketogenic diet/nutritional ketosis in neurological disorders.

### 4.1. Epilepsy

#### 4.1.1. Etiopathogenesis and Potential Role of Ketogenic Diet

Epilepsy is brain disease characterized by constant predispositions to generating electric impulses known as transient symptoms derived from hyperreaction of neurons or some brain areas [21,144]. The definition of epilepsy designates a specific frequency of seizures. Epilepsy is a consequence of many dysfunctions derived from environmental, genetic, physiological and pathophysiological factors [21,144]. The World Health Organization (WHO) reported four main reasons for epilepsy, that is trauma, central nervous system infections, cerebrovascular disorders and perinatal risk factors, however studies also suggest the involvement of factors such as oxidative stress [145,146,147] and channelopathies [148,149].

Epilepsy is one of the most cognizable neurological diseases. Treatment ends up with failure in one-third of cases. Although antiepileptic drugs tend to provide symptomatic relief, they do not modulate the underlying disease mechanism [144]. Therefore, it is important to implement alternative methods of treatment. It has been proven that the use of KD can be effective in the treatment of drug-resistant epilepsy [17,18,19].

Some types of epilepsy such as absence epilepsy (both early onset and childhood), myoclonic astatic epilepsy and focal epilepsy may be associated with GLUT1 deficiency syndrome [150,151]. Animal studies show that the ketogenic diet has been shown to attenuate the importance of glucose in brain metabolism by providing an alternative energy source in the form of ketone bodies [2]. Furthermore, the observed elevated levels of ketone bodies and glucose transporters in nutritional ketosis may have a beneficial effect on hypometabolism in the brain, thereby providing a potentially beneficial therapy for patients suffering from GLUT 1 deficiency syndrome [36].

Another disorder of glucose metabolism in the brain, hyperglycaemia, associated with insulin resistance or occurring episodically may increase the risk of epileptic seizures formation [152]. As mentioned earlier, KD prevents fluctuations in both fasting glycemia and level of insulin [75,76,77].

The previously described in vitro and in vivo animal studies show that ketone bodies can eliminate the effects of pathological processes such as neuroinflammation and oxidative stress. They also exert a protective effect on nerve cells by stimulating their regeneration, as well as activating ATP-sensitive potassium channels, resulting in decreased excitability of dentate granule neurons and networks [153].

A study of the effect of KD on infants with refractory epilepsy conducted by Xie et al. [126] showed that the microbiota of the studied group differed significantly from healthy infants. The ketogenic diet reduced detrimental bacteria from the *Enterobacteriaceae* family, such as *Escherichia* and *Salmonella*, as well as *Vibrio*. The diet also increased the number of *Bacteroidetes* and *Prevotella*, known to produce large amounts of SCFAs. A total of 64% of the study subjects showed a reduction in the frequency of seizures by 50%. Furthermore, Zhang et al. [128] reported increased amounts of *Bacteroidetes*, with a concomitant decrease in *Firmicutes* and *Actinobacteria* in patients with epilepsy who followed a KD for 6 months. Half of the study group showed a 50% reduction in seizures. The group not responding to KD therapy showed increased amounts of pathogenic bacteria present in the gut microbiota, such as *Clostridia*, *Bacteroidales* phylum-*Alistipes* and *Rikenellaceae*, and *Firmicutes* phylum-*Ruminococcaceae* and *Lachnospiraceae*.

Studies on rats have shown that KD increases levels of the inhibitory neurotransmitter GABA, thereby reducing neuronal hyperactivity and preventing seizures [52]. Interestingly, Olson et al. [136] demonstrated that both *Akkermansia muciniphila* and *Parabacteroides merdae* are significantly increased during KD treatment and they are essential for anticonvulsant activity. In addition, the observed higher GABA/glutamate ratio in the hippocampus of KD-fed mice compared to control-diet-fed mice was abolished by the administration of an antibiotic to the mice, and was again obtained after colonisation with *Akkermansia muciniphila* and *Parabacteroides merdae*.

#### 4.1.2. Indications for a Ketogenic Diet

In summary, over the past few decades, ketogenic nutritional therapy has become newly popular and has gained worldwide acceptance as an effective non-pharmacologic treatment for epilepsy. Several expert consensus guidelines on patient care have been published that attempt to define the mechanisms of action of this form of therapy and resolve doubts regarding its efficacy [154,155,156,157]. The researchers recommend implementing ketogenic diet therapies when two anticonvulsant drugs have been ineffective, and even earlier in certain syndromes, including GLUT1 deficiency syndrome, pyruvate dehydrogenase deficiency, epilepsy with myoclonic-atonic seizures, infantile spasms, tuberous sclerosis complex, children with gastrostomy tubes and Dravet syndrome [155].

The developed guidelines may allow the selection of the appropriate dietary therapy (ketogenic or less restrictive alternative diet) for the patient, in order to obtain the best possible treatment results while minimizing its side effects. However, there are still many questions that we do not know the answer to, such as potential risks to a foetus. Hopefully, future research lines in dietary ketogenic therapies in neurological disorders will provide answers.

#### 4.1.3. Clinical Data

The ketogenic diet is a well-established form of epilepsy treatment. Clinical trials and randomized controlled trials conducted over the past 7 years support the efficacy of the ketogenic diet in drug-resistant epilepsy (Table 1). Studies show that both children and adults [158,159,160,161,162,163,164,165,166,167,168,169,170] may experience improvements in seizure frequency, sometimes even achieving a complete absence of seizures.

### 4.2. Depression

#### 4.2.1. Etiopathogenesis and Potential Role of Ketogenic Diet

Depression is an increasingly diagnosed disease worldwide [171]. According to the World Health Organization (WHO), depression is the fourth most serious disease in the world and is predicted to become the most common CNS disease by 2030 [172]. The current increase of the incidence of depression results in serious consequences, since this disease not only is the main cause of suicide (the fourth leading cause of death in 15–29-year-olds), but also increases predisposition to other diseases [173]. It affects people of all ages, especially adolescents, young adults and the elderly [171]. Disorders of the functions of neurotransmitter systems (serotonin, norepinephrine, dopamine) are the pathophysiological basis of depression. On the other hand, external factors (stressors; some sociodemographic factors, such as female sex; postnatal depression; and traumatic experiences such as funeral, unemployment, mourning) can increase the risk and be the cause of the development of this disease [174].

It is presumed that one of the underlying causes of depression is impaired metabolism of tryptophan, a precursor to serotonin synthesis. Excellent sources of tryptophan include eggs, mozzarella cheese and pumpkin seeds, which can form the basis of a well-balanced ketogenic diet [175]. Furthermore, increased insulin sensitivity, as well as a change in the composition of the microbiome, contributes to alterations in neurotransmission. As previously mentioned, a study by Gupta et al. [68] on the effects of insulin on neurotransmission indicate that it may act by inhibiting the activity of MAO A and B, responsible for the degradation of serotonin, norepinephrine and dopamine, thereby increasing their levels.

Some evidence supports the involvement of other neurotransmitter systems in the aetiology of depression, such as glutamate, GABA, substance P and BDNF [171].

Moreover, the increase in GABA levels, the main inhibitory neurotransmitter, by this diet, as demonstrated in animal studies, may have a sedative effect or potentiate the effect of drugs whose mechanism relies on prolonging the opening of chloride channels in GABAergic receptors, such as benzodiazepines, enhancing their effect [52]. Certain probiotic bacteria colonising the large intestine (*Bifidobacterium*, *Lactobacillus*) have shown the ability to synthesise neurotransmitters such as GABA and serotonin, which may ameliorate disturbed neurotransmitter balance [176,177,178]. In addition, certain bacteria such as *Lactobacillus rhamnosus* reduce anxiety and depressive behaviour by altering GABA(B1b) and GABA(Aα) receptor expression in mice [179]. Kuwahara et al. [178] also reports that administration of lactic acid bacteria to rodents had a beneficial effect on BDNF levels, reducing anxiety and depressive behaviour. The ketogenic diet may reduce the number of these bacteria in the gut and probiotic supplementation is also worth considering.

The most abundant ketone body found in nutritional ketosis, β-HB, through inhibition of histone deacetylases increases BDNF secretion, which has neuroprotective and neuroregenerative effects which translates into improvement of mood [7].

#### 4.2.2. Clinical Data

Currently, the ketogenic diet has not been investigated in humans in the context of alleviating symptoms of depression, however KD has shown a positive effect on improving physical and mental well-being [180]. Moreover, animal studies indicate positive effects of the ketogenic diet on reducing anxiety and improving motor function [181,182]. These effects may also be due to reduced neuroinflammation and normalisation of neuronal excitability. All the above mechanisms suggest that KD may be a promising adjuvant therapy in patients suffering from depression.

### 4.3. Migraine

#### 4.3.1. Etiopathogenesis and Potential Role of Ketogenic Diet

Migraine is a common disorder affecting 10 to 20% of the population depending on the region of the world. Migraine is a chronic disease which significantly affects the quality of life. It is accompanied by bothersome headaches, vegetative disorders and hypersensitivity of various functional areas of the CNS. The causes of this disorder can be found in a specific combination of genetic and environmental factors. Currently it is not known which of these two factors plays a decisive role in the etiology of this disease. Each patient often struggles with an individual set of symptoms [183,184]. Nearly 25% of migraine sufferers experience specific, transient neurological symptoms known as migraine aura [184]. Migraine without an aura is defined as a clinical syndrome characterized by headache and following symptoms. Few patients also experience a prodromal phase, occurring a few hours or days before the headache, and/or a postdrome phase after the headache has subsided. Symptoms typical of these phases include hyperactivity, depressive states, cravings for determined foods, frequent yawning, fatigue and neck stiffness [185].

Taking into account the previous findings, it was concluded that migraine is conditioned by polygenetically dependent channelopathy, in which there is a predisposition to increased vasomotor activity [186]. Interestingly, despite the fact that KD prolongs the K_ATP_ channels opening in animal studies, which can cause migraine attacks both with and without aura [187], case studies reviewed by Gross et al. [143] indicate that patients using KD notice a reduction in migraine attacks frequency and severity.

Recent research shows that migraine is the result of impaired brainstem stimulation, which then involves the primary somatosensory region [188]. The brainstem plays an important role in generating migraine attacks and migraine with aura, which is an expression of the spread of cortical depression with accompanying hypoperfusion. Most likely, neurons in the brainstem area are depolarized, as a result of which the trigeminal nerve is activated (the main cause of meningeal vasodilation and neurogenic inflammation). Stimulation of this nerve can occur through the neuronal pathway as well as through neurotransmitters.

Inflammation and high concentrations of substance P cause arterial dilation and headache, which is the most characteristic symptom of a migraine attack [189]. Hypoglycaemia is shown to prolong the occurrence of cortical spreading depression [190]. Nutritional ketosis, by providing an alternative energy source, spares glucose and mitigates hypoglycaemia, which may result in a reduction of cortical spreading depression [191]. Additionally, many in vitro and animal studies have showed that redirection of the path of metabolism of selected amino acids towards increased synthesis of GABA-an inhibitory neurotransmitter [52], which balances excitatory and inhibitory neurotransmission, the anti-inflammatory effects [5,8,40,142], as well as the enhancing antioxidant systems [12,13], exhibited by ketone bodies may contribute to the efficacy of a low-carbohydrate diet in migraine.

For each patient, the “migraine threshold” is different. This balance between stimulation and inhibition of areas of CNS depends on a number of factors at the molecular level, such as ion channels function, magnesium levels and excitatory amino acids. Theoretical considerations and research, however, allow us to believe that KD may be effective in both the prevention and treatment of migraine [192].

#### 4.3.2. Clinical Data

To date, a clinical trial by di Lorenzo et al. [193] (Table 2) has shown that Very Low-Carbohydrate Ketogenic Diet (VLCKD) is effective in reducing migraine attacks. The number of migraine attacks decreased by -3.02 when using VLCKD compared with a very low-calorie non-ketogenic diet. However, exogenous administration of ketone bodies did not improve the patients’ condition [194].

### 4.4. Alzheimer’s Disease

#### 4.4.1. Etiopathogenesis and Potential Role of Ketogenic Diet

Generally speaking, the term dementia describes a decrease in cognitive abilities to degree that makes it impossible to perform daily activities. The most common form of dementia, especially in the elderly, is Alzheimer’s disease (responsible for nearly two-thirds of dementia cases in people over the age of 65) [195]. The risk of this disease increases with the age of the patient [196]. Experts predict that the number of cases of Alzheimer’s disease will be gradually increasing in the coming years [197]. Determining the beginning of the changes leading to the development of this disease is extremely complicated. This disorder leads to complete impairment of cognitive functions. It noticeably affects memory processes, understanding of uncomplicated issues, language proficiency and the ability to focus attention [195]. Symptoms in most cases begin with mild short-term memory loss, including recent memories [197].

In understanding the essence of this disease, it is important to determine the risk factors. Increasing age, serious head injuries, vascular disorders in the brain area, nicotinism or depression likely affect the rate of development of the disease [195]. Furthermore, genetic factors seem to play an important role in disease progression.

The primary pathological process underlying Alzheimer’s disease is the deposition of abnormal neuronal plaques and neurofibrillary tangles [198]. Plaques are defined as micro-changes in neurons that involve a core of Aβ surrounded by groups of enlarged axons. Beta-amyloid, under physiological conditions, derives from the amyloid precursor protein (APP). APP is split mainly by alpha and beta secretase. As a result of this process, small fragments of harmless Aβ arise. In the case of pathological changes, APP is split by gamma and beta secretase. As a result of this process, Aβ (42 peptides) is formed. Its accumulation and subsequent aggregation lead to the above-mentioned pathological changes. Beta-amyloid is deposited mainly in the vessels and the gray matter of the brain. In the described process, the genetic factors perform an important role, the gene responsible for the mentioned process of APP breakdown is located on chromosome 21, which is an important link in the family aetiology of Alzheimer’s disease [195]. Amyloid beta conglomerate is also deployed in the blood vessels of the brain leading to more or less extensive angiopathies that are responsible for extensive microbleeding all around the brain’s areas. Currently, it is believed that the deposition of amyloid plaques begins 20 years before the development of clinical manifestations [199].

The second important mechanism in the aetiology of Alzheimer’s disease is aggregation of neurofibrillary tangles composed of tau protein. Due to excessive aggregation of Aβ, hyperphostorilation of this structure occurs, which leads to its aggregation into larger, pathological conglomerates. It has been proven that these structures in the initial stages of the disease are present in the hippocampus. As the disease progresses, their presence can be found in neurons of the entire cerebral cortex [195]. The above-mentioned processes contribute to a significant reduction in the number of neurons in the cerebral cortex and specific subcortical regions. Animal studies have shown that KD reduces the volume of Aβ and tau protein aggregates, and reduces their toxicity [20,26,78]. However, this effect is limited to preventing the formation of new plaque. Thus, it can be speculated that KD may represent an interesting adjuvant therapy, resulting in slower disease progression and associated loss of cognitive function.

Furthermore, inflammatory processes initiated by the clusters of Aβ and tau protein can affect the expansion of the disease into new areas of the brain. Pro-inflammatory cytokines also play an important role in the destruction of brain tissue structures [138,139,140,141]. Oxidative stress and environmental factors may contribute to the development of the disease via disruption of the Hypothalamic–Pituitary–Adrenal (HPA) axis and insufficient removal of neurotoxic 4-hydroxynonenal [200]. Furthermore, the ApoEɛ4 allele responsible for late onset Alzheimer’s disease induces accelerated cellular ageing, as well as neuroinflammation and oxidative stress [201]. Studies performed on cell lines and animals provide evidence that the above-mentioned pathological phenomena may be alleviated by the application of KD, via limiting inflammation and oxidative stress [6,7,8,9,10,40,83].

#### 4.4.2. Medical Foods

There is no possibility of treating AD these days. The current approach to this disease is based on delaying the serious symptoms as long as possible [202]. Alleviation of that disease can be achieved both ways: pharmacological and non-pharmacological methods. The latter focus on cognitive training, physical activity and prescribed diets.

One such diet is the Mediterranean Diet. Extra-virgin olive oil (EVOO) contained in this diet seems to be crucial. According to Klimova and others [202], oleuropein, the secoiridoid contained in EVOO, may induce a neuroprotective effect, which indicates its potential use in the prevention of neurodegenerative diseases, in particular AD [202,203].

EVOO activity has been studied using animal mice models. Based on the results, it was determined that the active ingredients of EVOO improve the cognitive functions of mice’s brain by improvement of hippocampus synaptic activity and reduction of the accumulation of Aβ aggregate. EVOO may also alleviate the cytotoxic and neuroinflammatory consequences of the accumulation of Aβ aggregates [202,203]. Long-term supply of EVOO, excluding the impact on metabolism of Aβ aggregates, significantly affects reduction of phosphorylation of tau protein [203]. A diet rich in EVOO has been described as one which has no adverse effects such as cell death or neurodegeneration [202].

Another component of the Mediterranean diet are walnuts (*Juglans regia* L.) which, through the high content of antioxidants such as n-3 α-linolenic acid, juglone or tocopherol (vitamin E), are an important factor for the anti-neuroinflammatory effect of the diet. Enriching the diet of laboratory mice with walnuts resulted in improved memory and learning ability [204].

#### 4.4.3. Clinical Data

Many studies also indicate that insulin resistance may be a contributing factor in the development of neurodegenerative diseases [69]. The concomitant hyperglycaemia leads to changes in the brain, causing memory impairment. Weinstein et al. [205] have noted a reduction in gray matter volume in young people with hyperglycaemia, while Kerti et al. [206] have observed a reduction in hippocampal volume. Several studies have documented the association of impaired insulin signalling with protein Aβ [70,71] and thus Alzheimer’s disease [72,73,74]. Studies performed on Alzheimer’s patients indicate that KD normalises carbohydrate metabolism in the brain, reduces insulin levels, and increases insulin sensitivity [75,76,77]. The patients showed higher scores in tests of cognitive function, which indicates potential efficacy in neurodegenerative diseases [75,76]. The clinical studies conducted so far (Table 3) suggest that the ketogenic diet improves the cognitive performance of Alzheimer’s patients.

The significance of risk factors is still under the observation of researchers, but proper prevention and leading a healthy lifestyle are undoubtedly an important aspect in therapy and reducing the risk level of neurodegeneration. This is extremely crucial, considering the fact that the current pharmacotherapy strategy is based on alleviating the symptoms of the disease, but it does not contribute to the fight against its cause.

### 4.5. Parkinson’s Disease

#### 4.5.1. Etiopathogenesis and Potential Role of Ketogenic Diet

Parkinson’s disease is one of the most important neurodegenerative disorders next to Alzheimer’s disease. It occurs mainly in well-developed societies. Risk factors for PD include environmental toxins, drugs, genomic defects and cerebral vascular damage [211,212]. The risk of developing PD increases with the age of the patient. The risk of developing PD in people between the ages of 85 to 89 is 3.5%. In comparison, people under 60 years of age have a probability of development of PD at the level of 1% [212,213]. Diagnosis of PD takes place after the first psychomotor symptoms appear, which include muscle rigidity, resting tremors and motor retardation [214]. Bradykinesia is considered the primary diagnostic factor for the disease [215]. In addition to the typical motor symptoms, PD is accompanied by constipation, salivation, dysgraphia and extremely important cognitive and behavioural disorders, depression, sensory disturbances, sleep disorders, dementia and hallucinations [216]. The initial period of the disease is characterised by a postural defect and difficulties in walking. A freezing of gait, defined as a brief, episodic lack or restriction of foot progression despite the desire to walk is quite often noticed [217,218]. The main factor responsible for the development of the symptoms of the disease is the degeneration of neurons in the black matter, involved in the dopamine transmission of the nucleus basalis and the striatum [219]. Damage to these neurons leads to impaired dopamine transport which leads to dysfunction of neuronal circuits involving areas of the basal ganglia and motor cortex, which ultimately manifests as movement disorders [220]. Symptoms of PD only appear when the dopamine present in the basal nuclei and black matter drops to 20% of its maximum value [221]. In addition, one of the neuropathologies found in PD that may contribute to the death of dopaminergic neurons are Lewy bodies and Lewy neurites, composed of misfolded α-synuclein [222].

Currently, the primary drug for the treatment of PD is levodopa (L-DOPA), which has an effect on PD symptoms but no neuroprotective effect. It also appears that L-DOPA may favour the increased aggregation of α-synuclein, via the metabolite 5-S-cysteineldopamine, which induces oxidative stress in vivo, thereby promoting dopamine depletion [223]. Studies show that KD significantly improves the bioavailability of L-DOPA, which is associated with a reduction in dietary protein supply. The combination of symptom-control pharmacological treatment and KD may be effective in inhibiting further disease progression [78,121,224].

Kashiwaya et al. [225] conducted a study to elucidate the neuroprotective effects of β-HB. Heroin analogue, 1-methyl-4-phenylpyridinium, MPP(+), was used to induce black matter dopaminergic cell death by inhibiting the multi-enzyme mitochondrial NADH dehydrogenase complex, causing a Parkinson’s disease-like syndrome in cultures of midbrain neurons. One study confirmed previous findings that β-HB has neuroprotective effects on dopaminergic neurons [225,226,227]. This is related to increased mitochondrial respiration and increased ATP production. KBs also increase the efficiency of the mitochondrial respiratory chain by reducing oxygen free radicals [35].

One of the more recent potential therapies for PD is targeting K_ATP_ channels, the opening of which has been shown to have neuroprotective effects and to reduce neuronal excitability. However, it is presumed that activation of K_ATP_ channels located on GABAergic neurons may be one of the causes of PD development through inhibition of GABA_B_ receptors, which in turn stimulates glutamatergic terminals to secrete α-synuclein [228]. The effects of agonists or antagonists tested in animal models are inconclusive, suggesting that their impact on these channels is difficult to predict due to the high prevalence of K_ATP_ channels in the brain [228,229]. Nevertheless, in contrast to individual substances affecting K_ATP_ channels, the ketogenic diet acts simultaneously on multiple pathological processes, providing potentially better therapeutic efficacy.

#### 4.5.2. Clinical Data

In recent years, PD has been increasingly associated with alterations in the gut microbiota [113,121,230,231]. Alfonsetti et al. [113] reviewed microbiome composition and the effects of diet, probiotic, prebiotic and synbiotic administration on pathological processes occurring in the course of PD. Studies indicate that the microbiota of PD patients differs significantly from that of healthy individuals and is characterised by low numbers of *Prevotellaceae* and increased numbers of *Enterobacteriaceae* [232]. Alterations in the quality of the microbiota result in impaired intestinal permeability (i.e., “leaky gut”), which, through the LPS produced by bacteria, induces inflammatory processes and oxidative stress, thus promoting α-synuclein aggregation [233]. The ketogenic diet is known to reverse this ratio and thus increases *Prevotella* and decreases *Enterobacteriaceae* [131]. Changes in dietary habits (incorporating more omega-3 polyunsaturated fatty acids, probiotics, prebiotics and synbiotics into the diet) have been shown to have a beneficial effect on the course of the disease, through gut-sealing, anti-inflammatory, oxidative stress-relieving and BDNF upregulation effects [113].

A randomized controlled trial conducted in 2018 by Phillips et al. [234] for 8 weeks on 47 patients showed that both high-fat and low-fat diets had positive effects on motor and non-motor symptoms. However, the ketogenic diet exhibited greater improvements in non-motor aspects of ability to perform daily activities, i.e., urinary disturbances, pain, fatigue, or cognitive impairment compared to the low-fat diet.

## 5. Adverse Effects of the Ketogenic Diet

Clinical studies show that maintaining a ketogenic diet can be challenging for patients. Poor tolerance and lack of motivation may therefore provide causes for discontinuation of the diet [158]. Ketogenic diet modifications such as MAD were better tolerated among children with epilepsy [170], while alleviation of anxiety and cognitive activation were observed in the group using mostly MCTD (20/28 children) [159], suggesting that modifications of the ketogenic diet may be associated with greater compliance.

Since the groups using the ketogenic diet are mainly children suffering from epilepsy, its balance is a key element in determining a child’s proper growth. The diet should be well balanced to counteract the deficiencies brought about by abandoning a whole group of products rich in carbohydrates and other nutrients such as thiamin, folate, vitamin A, vitamin E, vitamin B6, calcium, magnesium, iron or vitamin K [235]. Patients may suffer from a deficiency of dietary fibres, which are essential for the proper functioning of the intestines, due to the exclusion of a certain group of products. A fibre deficiency leads to disorders in the proper absorption of nutrients, disruptions in the production of hormones responsible for satiety and a decrease in immunity [236].

The most difficult period for patients is the introduction to KD. During this time, the most common side effects of dieting are hypoglycaemia, dehydration and gastrointestinal disorders [237,238].

In the study conducted by Lin et al. [237], 57 out of 126 children experienced vomiting. Hypoglycaemia below 40 mg/dL occurred in 44 patients. In addition, constipation, irritability or negative mood changes were also observed. Six patients developed excessive ketosis with urinary ketone levels of 160 mg/dL, which manifested in facial flushing.

Various adverse gastrointestinal effects can occur during the use of KD. One of the main ones is constipation, which may result from an insufficient supply of fibre in the diet. Constipation can be managed by increasing the amount of fibre in the diet, performing enemas or administering polyethylene glycol [238].

One of the more common controversies surrounding the use of a high-fat diet is its effect on the lipid profile. Increased intake of fat-rich foods is the main cause of increased serum lipid fractions. In a study carried out by Cai et al. [239], it was shown that children suffered from hyperlipidaemia while taking KD, but at the same time this side effect was less frequent than the aforementioned gastrointestinal disorders. The mean cholesterol levels of the patients studied were slightly higher than before starting the diet.

There was a rapid response to the adverse changes regarding the increase in serum lipid fractions. Researchers Guzel et al. [166] proposed reducing dietary fat intake by 20–25% to improve lipid profile. The procedure was to eliminate products containing unsaturated fats and egg yolk sources, in addition, atorvastatin 10 mg daily was administered to inhibit endogenous cholesterol biosynthesis.

A study by Freeman et al. [18] also suggests a negative effect of the ketogenic diet on kidneys and the urinary system. In a group of 150 children, 3 of them had urate stones and 3 of them calcium oxalate or phosphate stones. It is therefore recommended that potassium citrate be used as a preventative measure throughout the diet [18,240].

## 6. Conclusions

Changes in eating habits can have a beneficial effect on the condition of our body, but also on the development and course of many diseases. This review provides evidence that the ketogenic diet may provide therapeutic benefits in patients with neurological problems associated with increased oxidative stress and neuro-inflammation or disruption in brain energy metabolism. The review of the scientific literature shows that KD could affect not only the progression of neurological disorders but also the course and outcome of their treatment. The effectiveness of KD has been proven in epilepsy and in other neurological diseases, such as depression, migraine, or neurodegenerative diseases e.g., AD and PD. KD should be also considered as an adjuvant therapeutic option in other neurological diseases.

## Figures and Tables

**Table 1:** Results of clinical trials and randomized controlled trials conducted over the past 7 years involving patients with epilepsy to determine the efficacy and tolerability of the ketogenic diet.

Author (Year)	Intervention Period	Diet	Group	Results
Kvernelnad et al. [158] (2015)	12 weeks	MAD	13 adults	>50% reduction of seizure frequency in 31% (4/13) adults
IJff et al. [159] (2016)	4 months	KD	28 (20 on MCT); 22 CAU ^a^	Cognitive activation, less anxiety and mood problems, increased productivity were observed in patients treated with the KD
Kim et al. [160] (2016)	6 months	KD	51	39% (20/51) KD patients had >50% seizure reduction, 31% (16/51) of them were seizure-free
		MAD	53	36% (19/53) had >50% reduction in seizures, 23% (12/53) were seizure free
Sharma et al. [161] (2016)	3 months	MAD	41 on MAD, 40 controls	56.1% (23/41) of the children on the diet had >50% seizure reduction, 14.6% (6/41) were seizure free compared to 5% (2/40) controls; 19.5% (8/41) had >90% seizure reduction
Ashrafi et al. [162] (2017)	4 months	KD (formula-based powder)	22	27.3% (6/22) had >90% reduction in seizures and 40.9% (9/22) had 50–90% reduction in seizures
Lambrechts et al. [163] (2017)	4 months	KD	26 KD	>50% reduction in seizure frequency in 50% (13/26) of KD, 11.5% (3/26) had >90% seizure reduction and another 11.5% (3/26) were seizure free
			22 CAU ^a^	18.2% (4/22) were responders; 9.1% (2/22) were seizure free and 4.5% (1/22) had >90% seizure reduction.
Baby et al. [164] (2018)	3 months	KD	54	59.4% (44/74) reported >50% seizure reduction. More than 90% reduction was noted in 33.7% children (25/74). 8.1% (6/74) became seizure free
	6 months		45	
	12 months		30	
Kverneland et al. [165] (2018)	12 weeks	MAD	24 on diet, 32 control group (habitual diet); adults	>25% seizure reduction among those who completed the intervention
Guzel et al. [166] (2019)	1 month	KD	369	65.8% (243/369) of the patients observed >50% decrease in seizure frequency; 35.5% (131/369) were seizure-free
	3 months		314	74.7% (235/314), of the patients observed >50% decrease in seizure frequency; 39.8% (125/314) were seizure-free
	6 months		225	70.6% (159/225) of the patients observed >50% decrease in seizure frequency; 38.2% (86/225) were seizure-free
	12 months		160	83.1% (133/160) of the patients observed >50% decrease in seizure frequency; 43.1% (69/160) were seizure-free
Bjurulf et al. [167] (2020)	7 months	KD with potassium citrate	22	>50% reduction in seizure frequency in 40.9% (9/22) patients supplementing potassium citrate and 27.6% (8/29) participants without potassium citrate
		KD without potassium citrate	29	
Gupta et al. [168] (2021)	12 weeks	LGIT	30	>50% reduction in seizure frequency in 73.3% (22/30) LGIT patients
		MAD	30	>50% reduction in seizure frequency in 43.4% (13/30) MAD patients
Lakshminarayanan et al. [169] (2021)	3 months	LGIT	20 on diet, 20 control group	30% (6/20) patients observed >50% reduction in seizure frequency
Poorshiri et al. [170] (2021)	6 months	KD	24	45.8% patients from KD group observed >50% decrease in seizure frequency
		MAD	11	45.5% from MAD group observed >50% decrease in seizure frequency

^a^ CAU: care as usual.

**Table 2:** Results of a clinical trial and a randomised controlled trial conducted in the last 7 years on migraine patients receiving Very Low-Carbohydrate Ketogenic Diet (VLCKD) or β-HB.

Author (Year)	Duration	Group	Intervention	Control	Results
Di Lorenzo et al. (2019) [193]	1 month	35 episodic migraine patients; 29 completed the study	VLCKD	very low-calorie non-ketogenic diet	reduction in migraine episodes
Putananical et al. (2022) [194]	12 weeks	41 episodic migraine patients	exogenous administration of β-HB	placebo	no clinically significant amelioration of migraine frequency or intensity

**Table 3:** Results of clinical trials and randomised controlled trials conducted on patients with Alzheimer’s disease over the past 7 years to determine the therapeutic efficacy of nutritional ketosis.

Authors	Duration	Group	Diet	Results
Torosyan et al. [207] (2018)	45 days	16	Caprylidene (ketogenic agent) administration	Increased blood flow in certain brain regions in patients lacking an APOEɛ4 allele
Ota et al. [208] (2019)	12 weeks	20	MCT based ketogenic formula	After 8 weeks, significant improvement in the immediate and delayed logical memory tests compared to their baseline scores were observed; at 12 weeks patients improved in the digit-symbol coding test and immediate logical memory test compared to their baseline scores
Fortier et al. [77] (2021)	6 months	83	ketogenic MCT drink	Free and cued recall verbal fluency, Boston Naming Test, and the Trail-Making Test improved significantly in the kMCT group compared to placebo
Myette-Côté et al. [209] (2021)	6 months	39	ketogenic MCT drink	No clinically relevant adverse effect on the blood markers. After intervention plasma IL-8 significant increase have been observed
Philips et al. [210] (2021)	two 12-week treatment periods	26	KD	Improved daily function and quality of life

## Data Availability

Not applicable.
